# ROS Production and Scavenging under Anoxia and Re-Oxygenation in *Arabidopsis* Cells: A Balance between Redox Signaling and Impairment

**DOI:** 10.3389/fpls.2016.01803

**Published:** 2016-12-01

**Authors:** Annalisa Paradiso, Sofia Caretto, Antonella Leone, Anna Bove, Rossella Nisi, Laura De Gara

**Affiliations:** ^1^Dipartimento di Biologia, Università degli Studi di Bari Aldo MoroBari, Italy; ^2^Consiglio Nazionale delle Ricerche, Istituto di Scienze delle Produzioni AlimentariLecce, Italy; ^3^Food Science and Nutrition Unit, Department of Medicine, Università Campus Bio-Medico di RomaRoma, Italy

**Keywords:** anoxic stress, antioxidant defenses, bioreactor, hydrogen peroxide, nitric oxide, reactive oxygen species

## Abstract

Plants can frequently experience low oxygen concentrations due to environmental factors such as flooding or waterlogging. It has been reported that both anoxia and the transition from anoxia to re-oxygenation determine a strong imbalance in the cellular redox state involving the production of reactive oxygen species (ROS) and nitric oxide (NO). Plant cell cultures can be a suitable system to study the response to oxygen deprivation stress since a close control of physicochemical parameters is available when using bioreactors. For this purpose, *Arabidopsis* cell suspension cultures grown in a stirred bioreactor were subjected to a severe anoxic stress and analyzed during anoxia and re-oxygenation for alteration in ROS and NO as well as in antioxidant enzymes and metabolites. The results obtained by confocal microscopy showed the dramatic increase of ROS, H_2_O_2_, and NO during the anoxic shock. All the ascorbate-glutathione related parameters were altered during anoxia but restored during re-oxygenation. Anoxia also induced a slight but significant increase of α-tocopherol levels measured at the end of the treatment. Overall, the evaluation of cell defenses during anoxia and re-oxygenation in *Arabidopsis* cell cultures revealed that the immediate response involving the overproduction of reactive species activated the antioxidant machinery including ascorbate-glutathione system, α-tocopherol and the ROS-scavenging enzymes ascorbate peroxidase, catalase, and peroxidase making cells able to counteract the stress toward cell survival.

## Introduction

Plants being aerobic organisms need oxygen as an essential substrate for energy production. However, oxygen can fall to low concentrations in many plant tissues because of environmental factors such as flooding or waterlogging which reduce the external oxygen concentration. As well, the poor distribution efficiency for oxygen through plant organs or high rates of cellular metabolism in dividing meristem cells can induce a severe drop of plant internal oxygen concentrations. Oxygen deprivation stress in plant cells includes three different states characterized by different oxygen concentrations: hypoxia, anoxia, and re-oxygenation. The responses to oxygen deprivation include various alterations of plant metabolism aimed at plant survival such as energy preservation, reduction of respiration, and induction of fermentation which occurs when oxygen levels are close to zero (Geigenberger, 2003). Microarray analyses revealed several genes highly responsive to oxygen deficiency in different plant species (Mustroph et al., 2010). Recently, an oxygen sensing mechanism involving specific transcription factors, able to trigger the plant molecular response to hypoxia was identified (Licausi et al., 2011; Sasidharan and Mustroph, 2011).

In addition, a mechanism of ROS signaling, involving the production of hydrogen peroxide and acting under oxygen deprivation, was found to be linked to oxygen sensing (Gonzali et al., 2015).

Anatomical and morphological changes such as aerenchyma formation also occur in several plant species to cope with frequent flooding. This adaptation, aimed at improving gas exchange, is mediated by ROS, in particular hydrogen peroxide (Steffens et al., 2011).

It has been reported that in plants during the transition to hypoxia/anoxia from normoxic conditions or during re-oxygenation, ROS and NO can be excessively produced. Paradoxically plant responses to oxygen deprivation appear to include the production of hydrogen peroxide or other ROS (Bailey-Serres and Chang, 2005; Blokhina and Fagerstedt, 2010; Irfan et al., 2010). In these conditions, an impairment of redox balance occurs that requires the activation of defense pathways involving redox metabolites and related enzymes responsible to counteract ROS production and to recover redox homeostasis. The plant redox defense system is represented by several molecules, both hydrophilic and lipophilic, exerting their effects in different cell compartments such as ASC, glutathione and tocopherols. In addition, enzymes able to directly eliminate ROS excesses can be activated: superoxide dismutases, CATs, and PODs using different electron donor as well as enzymes responsible for the recycling of redox pairs, in particular ASC and glutathione.

Several studies have been carried out on the redox balance of plant tissues under oxygen deprivation stress, however, variable responses have been reported for different plant species and different experimental conditions; in particular, the levels of antioxidant molecules and the activity of antioxidant regenerating enzymes did not show equal trends (Blokhina et al., 2003). The resistance of a plant species/variety to anoxic stress, the physiological conditions of the exposed plant, as well as stress intensity are factors affecting plant resilience.

Establishing experimental conditions, which make plant cells uniformly exposed to a controlled oxygen supply, is not simple to achieve. Cultivating plant cells in a bioreactor system makes it possible to control physicochemical parameters to be homogeneous throughout the culture. Furthermore, the heterogeneity of oxygen availability often occurring in different cells or tissues in the same organ is avoided in suspension cells. Therefore, the use of bioreactors can be a suitable system for studying plant cell response to oxygen deprivation. In a previous work, *Arabidopsis* cell cultures subjected to hypoxia (static flasks) or anoxia (bioreactor) revealed a defense response involving an increase of the levels of H_2_O_2_ in the medium and the antioxidant metabolite α-tocopherol (Nisi et al., 2010). To shed more light on this response, *Arabidopsis* cell suspension cultures grown in a stirred bioreactor were subjected to a severe anoxic stress and analyzed during anoxia and re-oxygenation for the alteration in ROS, H_2_O_2_, and NO as well as in antioxidant enzymes and metabolites.

## Materials and Methods

### Cell Cultures

Cell suspension cultures of *Arabidopsis thaliana* L., Heynh., ecotype Landsberg were maintained in MS (Murashige and Skoog, 1962) medium supplemented with 30 g l^-1^ sucrose, 0.5 mg l^-1^ NAA (naphthaleneacetic acid), 0.05 mg l^-1^ Kinetin. Cell suspensions were subcultured in 500 ml flasks at 15-day intervals by inoculating 2 ml of packed cell volume in 50 ml of fresh medium.

MS medium (3.150 l) containing 30 g l^-1^ sucrose, 0.5 mg l^-1^ NAA, 0.05 mg l^-1^ Kinetin was inoculated with 350 ml of 14-day-old shake flask suspension in a 5.0 l stirred bioreactor (BioFlo 110, New Brunswick Scientific, Edison, NJ, USA). Cultivation was performed at 25°C, pH 5.6 and 80 rpm agitation speed, under continuous fluorescent white light (50 μmol photons m^-2^ s^-1^). Before the anoxic stress, cells were cultivated under 20% dissolved oxygen (DO) of air saturation, automatically obtained by the gas mix controller (New Brunswick Scientific, Edison, NJ, USA).

### Experimental Design

*Arabidopsis* cells grown in a stirred bioreactor for 8 days under 20% DO were subjected to anoxia for 4 h, by stopping aeration and fluxing with nitrogen into the vessel (0.01% DO). During the whole period of treatment, the bioreactor was maintained in the dark. Thereafter they were re-oxygenated by restoring the previous aeration conditions for 20 h. All the analyses were performed on samples taken at different times: T0, normoxia; T1 and T2, anoxia for 2 and 4 h, respectively; T3 and T4, 2 and 20 h after re-oxygenation, respectively. T1 and T2 samples were collected under nitrogen flux and immediately used for avoiding re-oxygenation.

### Cell Viability

*Arabidopsis* cell suspension cultures were stained with the Evans Blue dye and cell death was determined by spectrophotometric analysis according to Carimi et al. (2003). For each time, three independent experiments were performed with each assay done in triplicate. Dead cells were also analyzed by light microscopy according to de Pinto et al. (1999). For each treatment, 500 cells were examined.

### Ascorbate and Glutathione Analyses

For ASC and glutathione determination, 0.3 g cells were homogenized with cold 5% metaphosphoric acid at 4°C at 1:3 ratio (w/v) in order to obtain a deproteinized extracts. After centrifugation at 20000 g for 20 min, the supernatants were collected and used for ASC and GSH analysis according to Paradiso et al. (2006).

### Extraction and Analysis of Tocopherols

Extraction and analysis of tocopherols were carried out as previously described (Caretto et al., 2002). Briefly, the method consisted of an alkaline hydrolysis (potassium hydroxide 60%) followed by extraction with *n*-hexane-ethyl acetate (9:1). Chromatography separation was performed by using a Beckman HPLC Analytical System. A RPC18 Beckman Ultrasphere column was used with methanol (98%) as the mobile phase. Two programmable detectors, an ultraviolet-visible spectrophotometer (set at *λ*: 290 nm) and a spectrofluorimeter (*λ* excitation: 289 nm; *λ* emission: 325 nm) were connected in series to determine tocopherols. The tocopherol content was calculated by means of standard calibration curves. Each experiment was carried out with at least three replicates.

### ROS, NO, and H_2_O_2_ Detection by Confocal Laser Scanning Microscopy (CLSM)

A confocal laser scanning microscope system, CLSM (Zeiss LSM Pascal, Carl Zeiss Inc., Germany), equipped with He-Ne and Ar lasers and coupled to Axiovert 200 inverted microscope (Zeiss, Germany) was used to detect ROS, NO, and H_2_O_2_ in anoxia-treated and untreated *Arabidopsis* cells by using specific fluorescent probes.

Reactive oxygen species were detected by using 25 μM carboxy-H_2_DCFDA in DMSO, from Image-iT^TM^ LIVE Green ROS Detection Kit (Molecular Probes, Invitrogen, Ltd., Paisley PA4 9RF, UK) following the manufacturer instructions. Two hundred microliters of anoxia-treated cell cultures, at different times, and the relative controls, were incubated for 30 min at 25**°**C, in darkness, with 25 μM carboxy-H_2_DCFDA probe. Then cell suspensions were centrifuged at 2000 × *g*, washed twice with 1 ml of fresh medium and finely suspended in 200 μl of medium. Cells were immediately observed by CLSM.

Hydrogen peroxide (H_2_O_2_) presence was detected by DHR probe (Life Technologies, Carlsbad, CA, USA). Two hundred and fifty microliters of anoxia-treated and control cells were incubated 10 min with 1 μl of DHR, in the darkness. The medium was then removed and the cells were washed twice with fresh medium and immediately imaged by CLSM. The specificity of the staining for H_2_O_2_ was tested in a parallel experiment by adding 30 U ml^-1^ of CAT before the addition of DHR.

For NO detection a 250 μl of cell culture sample, were incubated for 10 min at 25**°**C, in darkness, with 1 μl of DAF-2 DA (Calbiochem-Novabiochem) diluted in medium, then cells were centrifuged at 2000 × *g*, washed twice in fresh medium and immediately imaged by CLSM.

Carboxy-H_2_DCFDA, DAF-2 DA, and DHR probes were excited with the 488 nm line, and filtered through a 505–530 nm band pass filter, fluorescence was displayed as green false color. The second channel collecting emission beyond 650 nm by a long band-pass filter (excited at 488 nm laser line) was used to collect the autofluorescence of chlorophylls and the highly excited R123, and was displayed as red false-color. Background staining, routinely negligible, was controlled with unstained cells, which showed only the red autofluorescence of chloroplasts (data not shown). Confocal images were recorded using Plan-Neofluar 20×/0.5 and Plan-Neofluar 40×/0.75 objectives. A minimum of nine randomly taken fields, per treated and not treated cell cultures, was recorded. LSM Pascal 5 software was used to record and match confocal images.

### Enzyme Assays

Cells were ground in liquid nitrogen with a mortar and pestle. Five volumes of a buffer containing 50 mM Tris-HCl (pH 7.5), 0.05% (w/v) cysteine, 0.1% (w/v) BSA, were added just as the last trace of liquid N_2_ disappeared. The thawed mixture was then ground and centrifuged at 20000 g for 15 min. The supernatant was used for spectrophotometric analysis.

Cytosolic APX (L-ascorbate: hydrogen peroxide oxido-reductase, EC 1.11.1.11) activity was measured according to Locato et al. (2009) by following the H_2_O_2_-dependent oxidation of ASC at 290 nm in a reaction mixture containing 0.1 M Tris-acetate buffer, pH 6.4, 350 μM ASC, 170 μM H_2_O_2_, 50–100 μg protein. The non-enzymatic H_2_O_2_-dependent oxidation of ASC, as well as the oxidation of ASC, not dependent upon H_2_O_2_ addition, was subtracted.

Dehydroascorbate oxido-reductase (glutathione: DHAR, EC 1.8.5.1), MDHAR (NADH: monodehydroascorbate radical oxido-reductase, EC 1.6.5.4), and CAT (hydrogen peroxide: hydrogen peroxide oxido-reductase, EC 1.11.1.11) were assayed according to Paradiso et al. (2012). Peroxidase activity (POD EC 1.11.1.7) was measured following the oxidation of 3,3′,5,5′-Tetramethylbenzidine (TMB) at 652 nm (𝜀 = 26,9 mM^-1^ cm^-1^), according to Ferrer et al. (1990).

Protein content was determined according to Bradford (1976) using bovine serum albumin as standard. All enzyme activities were measured using a Beckman (Fullerton, CA, USA) DU 7000 spectrophotometer.

### Total RNA Extraction and Semi-Quantitative RT-PCR

Total RNA was isolated from *Arabidopsis* cells using the RNeasy plant minikit (QUIAGEN S.p.A., Milan, Italy) according to the supplier’s recommendations. Residual DNA was removed from the RNA samples using a DNA-*free* kit (AMBION, Inc., Austin, TX, USA). Synthesis of cDNA was performed from 2 μg total RNA with 10 μM random primers (AMERSHAM Biosciences Europe GMBH, Milan, Italy), utilizing an Omniscript Reverse Transcriptase kit (QUIAGEN S.p.A., Milan, Italy) according to the supplier’s recommendations. PCR reactions were performed with specific primers for APX1 (X59600, 5′-GGACGATGCCACAAGGATAG-3′ and 5′-GGTTGCGATTTGAACACAT-3′); APX2 (X98275, 5′-ATTGCCGTTAGGCTTCTTGA-3′ and 5′-TACCAACCGACAAGGCTCTT-3′); APX6 (AV555486, 5′-CTGCTGGTGTGCTTCGTTTA-3′ and 5′-TTGAAAAACCATGGACGTCA-3′) and 18S rRNA (AJ236016, 5′-CATGATAACTCGACGGATCG-3′ and 5′-GAAGGCCAACGTAATAGGAC-3′). 18S rRNA was used as an internal control in order to normalize each sample for variations in the amount of initial RNA. For semi-quantitative RT-PCR, the cycle number in the linear range was empirically determined. These were analyzed on 1.5% agarose gel containing 0.5 μg mL^-1^ ethidium bromide.

### Statistical Analysis

Experiments were repeated five times and a statistical analysis was performed. Data were analyzed by one-way analysis of variance (ANOVA).

## Results and Discussion

Several studies have reported ROS generation in response to oxygen deprivation or re-oxygenation in plants. The ROS, in particular H_2_O_2_, generated during stress have been implicated as triggers of signaling pathways influencing the expression of nuclear encoded genes which may initiate acclimation processes contributing to stress tolerance (Banti et al., 2013). In this study, a rise in reactive oxygen and nitrogen species during anoxia and the activation of antioxidant system during re-oxygenation is shown in *Arabidopsis* cell suspensions grown in a bioreactor, which allowed the close control of oxygen levels.

### Cell Viability

At different time intervals cell samples of *Arabidopsis* cell cultures subjected to anoxia and re-oxygenation were taken and analyzed for cell viability. Since no significant differences were observed between control and treated cells, cell viability was not affected by anoxia followed by re-oxygenation (data not shown).

### ROS, H_2_O_2_, and NO Detection by CLSM

The production of the main reactive species in *Arabidopsis* cell cultures was evaluated during normoxia, anoxia, and re-oxygenation by using fluorescent probes able to detect ROS, H_2_O_2_, and NO. Only the spotted red fluorescence, due to the autofluorescence of chloroplast chlorophylls was observed in cells incubated in the absence of the fluorescent probes as control (data not shown).

**FIGURE 1:**
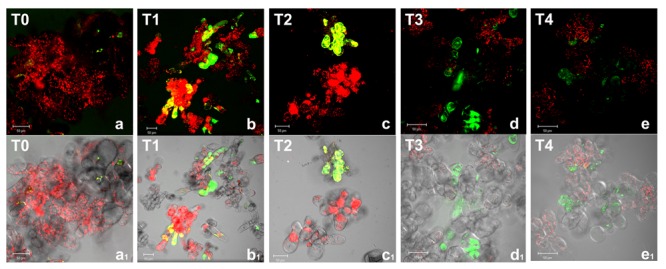
**Reactive oxygen species detection by confocal laser scanning microscopy in *Arabidopsis thaliana* cell suspension cultures.** Cells were grown in bioreactor in normoxic conditions (T0), subjected to anoxia 2 h (T1) or 4 h (T2) and re-oxygenation 2 h (T3) or 20 h (T4). Merged two-color confocal images **(a–e)** of the green and the red channel show autofluorescence of chloroplasts (red false color) and nonspecific ROS (green false color) detected by carboxy-H_2_DCFDA probe. In **(a_1_–e_1_)** the confocal microscopy images are digitally merged with the transmitted light images for displaying the number of labeled cells in the total cell population. Excitation wavelength 488 nm line, emission was detected at 505–530 nm band pass filter and 650 nm long band-pass filter. Scale bar = 50 μm.

Reactive oxygen species production was detected in *Arabidopsis* cell cultures under different oxygen levels by using carboxy-H_2_DCFDA (**Figure 1**). This probe, when deacetylated by intracellular esterases and oxidized by ROS, emits bright green fluorescence. In normoxic conditions (T0), very few cells showed faint fluorescence as defined single spots, likely due to the basal metabolism. During anoxia (T1 and T2), a diffuse green fluorescence was observed in an increasing number of cells indicating that ROS increase affected the whole cell, thus suggesting the occurrence of an oxidative stress, an usual event under different stress conditions. During re-oxygenation (T3 and T4), a reduction of both fluorescent cell number and fluorescence intensity was observed. Twenty hours after the end of anoxia (T4), cells seemed to restore completely the normoxic conditions being ROS detectable as spotted green-fluorescence.

**FIGURE 2:**
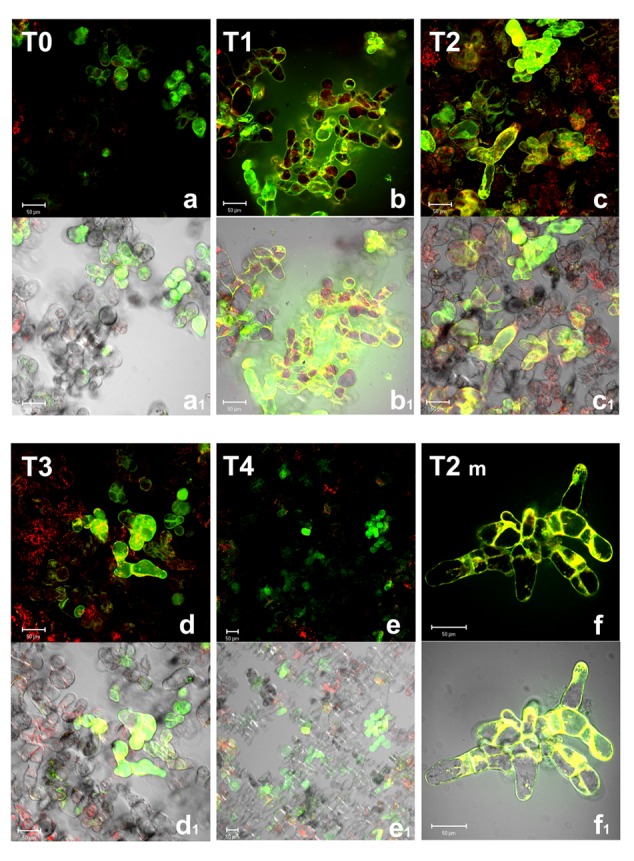
**Hydroxyl peroxide (H_2_O_2_) detection by confocal microscopy in *A. thaliana* cell suspension cultures.** Cells were grown in bioreactor in normoxic conditions (T0), subjected to anoxia 2 h (T1) or 4 h (T2) and re-oxygenation 2 h (T3) or 20 h (T4). T2m represents a magnification of T2 cells. Merged two-color confocal images **(a–f)** of the green and the red channel show H_2_O_2_ (green false color) detected by DHR probe (R123), and autofluorescence of chloroplasts (red false color). In **(a_1_–f_1_)**, the confocal microscopy images are digitally merged with the transmitted light images for displaying the number of labeled cells in the total cell population. Excitation wavelength 488 nm line, emission was detected at 505–530 nm band pass filter and 650 nm long band-pass filter. Scale bar = 50 μm.

Among ROS, the production of H_2_O_2_ was reported to increase in several abiotic stress conditions including oxygen deprivation (Blokhina and Fagerstedt, 2010). This ROS is of particular relevance for its relative stability under different physiological conditions and its capability to cross biological membranes. Indeed, it is well known that alterations in H_2_O_2_ are part of the redox signaling pathways activating defense responses in all aerobic organisms. H_2_O_2_ accumulation was then investigated by using 1,2,3-dihydrorhodamine (DHR), a probe which in the presence of H_2_O_2_ is oxidized to its fluorescent form R123 (Hensley et al., 2003).While DHR passively diffuses across plasma membrane, the charged fluorescent rhodamine-123 cannot cross being trapped in cellular compartments (Kinsey et al., 1987; Henderson and Chappell, 1993). Confocal images (**Figure 2**) show that, in normoxic conditions (T0), only few *Arabidopsis* cells displayed green fluorescence, due to the basal metabolism. Under anoxic conditions, H_2_O_2_ levels strongly increased. It is worth noting that during anoxia cell boundaries appeared to be particularly involved in H_2_O_2_ production (T1, T2, and T2m). Several enzymatic systems are responsible for O_2_^-^/H_2_O_2_ production at plasma membrane/cell wall interface. Among these, NADPH oxidase and apoplastic superoxide dismutase are well known enzymes participating in ROS overproduction in a plethora of environmental biotic and abiotic stresses, including low oxygen (Pucciariello and Perata, 2016). Specific PODs and amine oxidases also contribute to cell wall O_2_^-^/H_2_O_2_ production at least in some cases of stress conditions (Hurkman and Tanaka, 1996; Angelini et al., 2008). At the end of anoxic stress (T2), most *Arabidopsis* cells (about 70–80%) appeared green or yellow-green fluorescent. An increase of H_2_O_2_ during anoxia is coherent with our previous results indicating a rise for H_2_O_2_ released in the culture medium during anoxia (Nisi et al., 2010). During re-oxygenation (T3 and T4) H_2_O_2_ production decreased, only few cells being green fluorescent. The cellular compartment involved in H_2_O_2_ production also seems to be different being the plasma membrane/cell wall interface probably less relevant and intracellular production more relevant during re-oxygenation than in anoxia. This is consistent with the recovery of mitochondrial electron flow, previously blocked by oxygen deprivation and with a role for H_2_O_2_ in metabolic signaling within cells.

**FIGURE 3:**
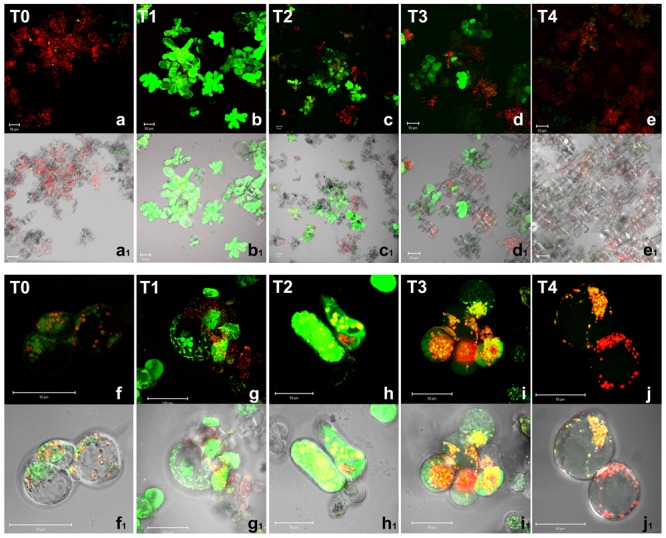
**Nitric oxide detection by confocal microscopy in *A. thaliana* cell suspension cultures.** Cells were grown in bioreactor in normoxic conditions (T0), subjected to anoxia 2 h (T1) or 4 h (T2) and re-oxygenation 2 h (T3) or 20 h (T4). Merged two-color confocal images **(a–j)** of the green and the red channel show autofluorescence of chloroplasts (red false color) and NO (green false color) detected by DAF-2 DA probe. In **(a_1_–j_1_)**, the confocal microscopy images are digitally merged with the transmitted light images for displaying the number of labeled cells in the total cell population and the cellular localization of the fluorescent probe. **(f–j)** and **(f_1_–j_1_)** are at higher magnification. Excitation wavelength 488 nm line, emission was detected at 505–530 nm band pass filter and 650 nm long band-pass filter. Scale bar = 50 μm.

A tight interplay between ROS and reactive nitrogen species has been reported in stress signaling; NO has also been reported to be induced by anoxic conditions (Stöhr and Stremlau, 2006). NO production was then analyzed in *Arabidopsis* cells by CLSM using DAF-2 DA, a probe highly specific for NO (Kojima et al., 1998; Nakatsubo et al., 1998). Because of the short half-life (5–15 s) of NO, the detected fluorescence indicates the amount and the cell localization of NO production at a given time (**Figure 3**).

In normoxia conditions (T0), a basal level of NO production was detected as green fluorescent spots only in few scattered cells (**Figures 3a,a**_1_); fluorescence was clearly visible in round shaped endocellular organelles; according to merge picture, almost no production was evident in chloroplasts (**Figures 3f,f**_1_). The subcellular site of NO synthesis in plants is still under debate. Among the subcellular compartments suggested to be responsible for such biosynthesis, peroxisomes are probably the best-characterized (Corpas et al., 2013). However, under hypoxic conditions, NO production was also suggested to occur in other compartments such as the cytosol and organelles including mitochondria, which were shown to be a significant source of NO in various organisms including plants (Gupta et al., 2011). During the anoxic stress, a great increase of NO production was observed. It is worth noting that at 2 h anoxia, most cells appeared green fluorescent. At this early stage of anoxia (T1), *Arabidopsis* cells showed a diffuse green fluorescence due to NO within the whole cell volume (**Figures 3b,b_1_,g,g_1_**). The pre-incubation with the NO scavenger 2-(4-Carboxyphenyl)-4,4,5,5-tetramethylimidazoline-1-oxyl-3-oxide confirmed that green florescence was actually due to NO (data not shown). In T2, ∼60–80% of *Arabidopsis* cells displayed a positive reaction to the probe DAF-2 DA (**Figures 3c,c_1_,h,h_1_**). During the early recovery period 2 h after re-oxygenation NO was still produced in a relevant number of cells (T3, **Figures 3d,d_1_,i,i_1_**) even if at lower levels compared to T1. After 20 h of re-oxygenation (T4), cells restored the baseline conditions of NO production with very few cells showing faint green fluorescence (**Figures 3e,e_1_**), which was evident as small dots (**Figures 3j,j_1_**). Taken together these results indicate that oxygen shortage induced an oxidative burst. ROS and NO production seemed to be particularly high during the 1st hours of anoxia. During re-oxygenation, ROS, and NO were still produced. Under our experimental condition, almost 20 h were required in order to recovery redox homeostasis.

The transient increase of ROS, H_2_O_2_, and NO in *Arabidopsis* cultured cells during oxygen deprivation was likely able to trigger cell response for allowing the cells to overcome the anoxic stress. Consistently, in spite of such increases, no decrease in cell viability was induced.

### ROS Scavenging Systems

In plant cells a complex network of redox metabolites and enzymes guarantees redox homeostasis. This network includes several ROS scavenging enzymes (CAT, PODs) and a network of antioxidant molecules, such as ascorbic acid, glutathione, α-tocopherol, that interact with ROS directly or in reactions carried out by antioxidant enzymes such as APX (Noctor and Foyer, 1998; Apel and Hirt, 2004). In addition, a whole array of enzymes is needed for the regeneration of the active forms of the antioxidant molecules (MDHAR, DHAR, and GR).

The importance of the ASC-glutathione system for the detoxification of hydrogen peroxide has been well characterized in different stress conditions (Locato et al., 2009; de Pinto et al., 2015) but little detailed information is available about its action in anoxic stress.

**FIGURE 4:**
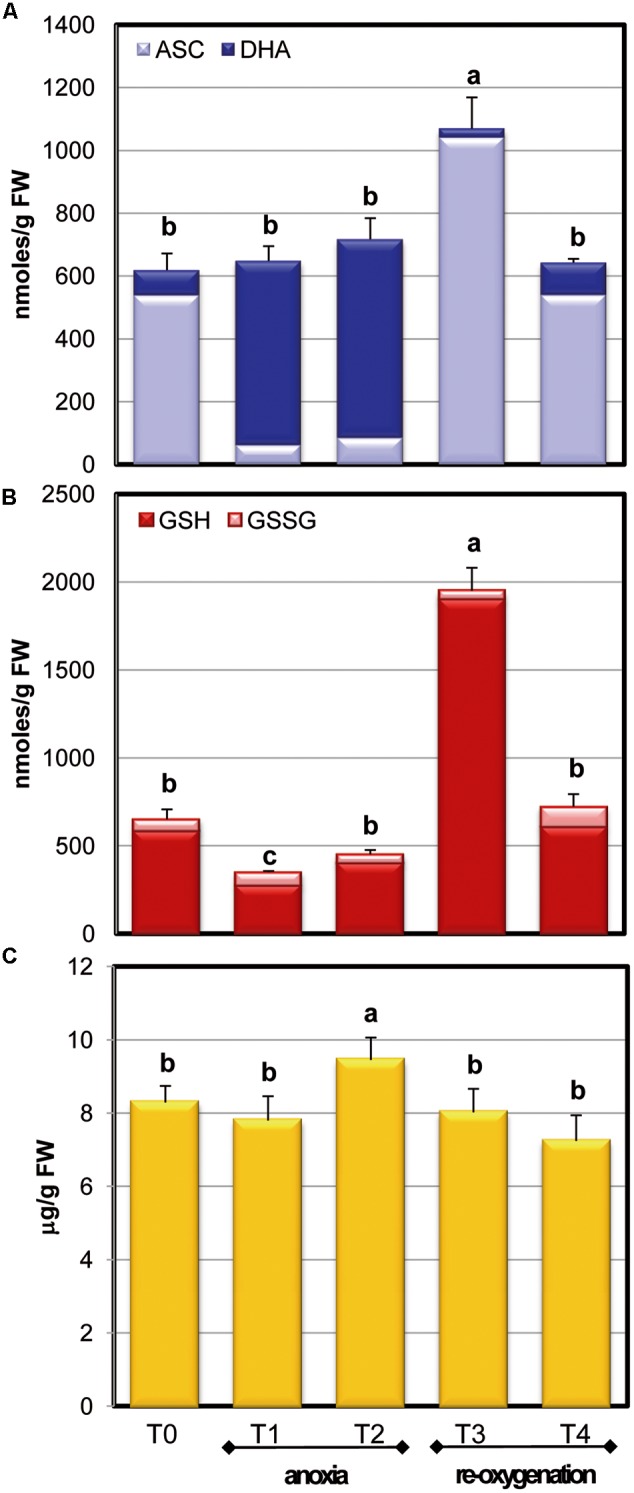
**Effects of anoxia and re-oxygenation on ASC pool, GSH pool, and α-tocopherol in *A. thaliana* cell suspension cultures.** ASC +DHA **(A)** and GSH+GSSG **(B)** contents are expressed as nmoles/g FW; α-tocopherol **(C)** as μg/g FW. Data represent the means (±SE) of five experiments. Different letters represent values which are statistically different (by one-way Anova test).

The behavior of ASC pool during anoxia indicates that no significant alteration occurred when the total ASC plus DHA is considered (**Figure 4A**). In contrast, the redox state (ASC/ASC + DHA) was severely affected by the variations of oxygen concentration. After 2 h of anoxia (T1), a significant increase of the oxidized form (DHA) was observed, resulting in a decrease of the redox state (0.82 ± 0.15 and 0.11 ± 0.042 at T0 and T1, respectively) that remained unchanged up to the end of anoxia (T2). This confirms the strong redox impairment occurring during anoxia. After 2 h of re-oxygenation (T3), however, the total content of ASC increased significantly, reaching almost doubled values compared to T0 cells. T4 cells showed ASC levels and redox state fully comparable to those of T0 cells (de Pinto et al., 2006; Munné-Bosch et al., 2013).

The anoxic treatment caused a significant decrease of total glutathione content during the first 2 h of anoxia (T1) (**Figure 4B**), while glutathione pool increased again to the T0 value in the following 2 h of anoxia. Differently from ASC, GSH redox state remained unchanged during oxygen concentration changes, with a ratio of more than 90% in reduced form. After re-oxygenation (T3), GSH content increased over threefold: the increase of ASC and glutathione pool, occurring at this stage, emphasized that the cells were responding to an extraordinary stress situation by enhancing their redox molecules. At T4 GSH returned to control values, similarly to what occurred for ASC, thus showing that cells were able to restore the physiological redox balance.

The levels of the lipophilic antioxidant metabolite α-tocopherol were also measured during normoxia, anoxia, and re-oxygenation (**Figure 4C**). In these experimental conditions, α-tocopherol showed a slight but significant increase at T2 phase and a slow decrease after re-oxygenation until reaching values similar to control cells (T4). In plants, α-tocopherol is known to increase in several environmental constraints involving oxidative stress (Munné-Bosch, 2005) thus an increase during anoxia could be expected. On the other hand, since ASC is involved in the process of regeneration of tocopherols, such regeneration during anoxia might likely contribute to the observed decrease in the ASC/DHA redox state.

### Antioxidant Enzymes

**FIGURE 5:**
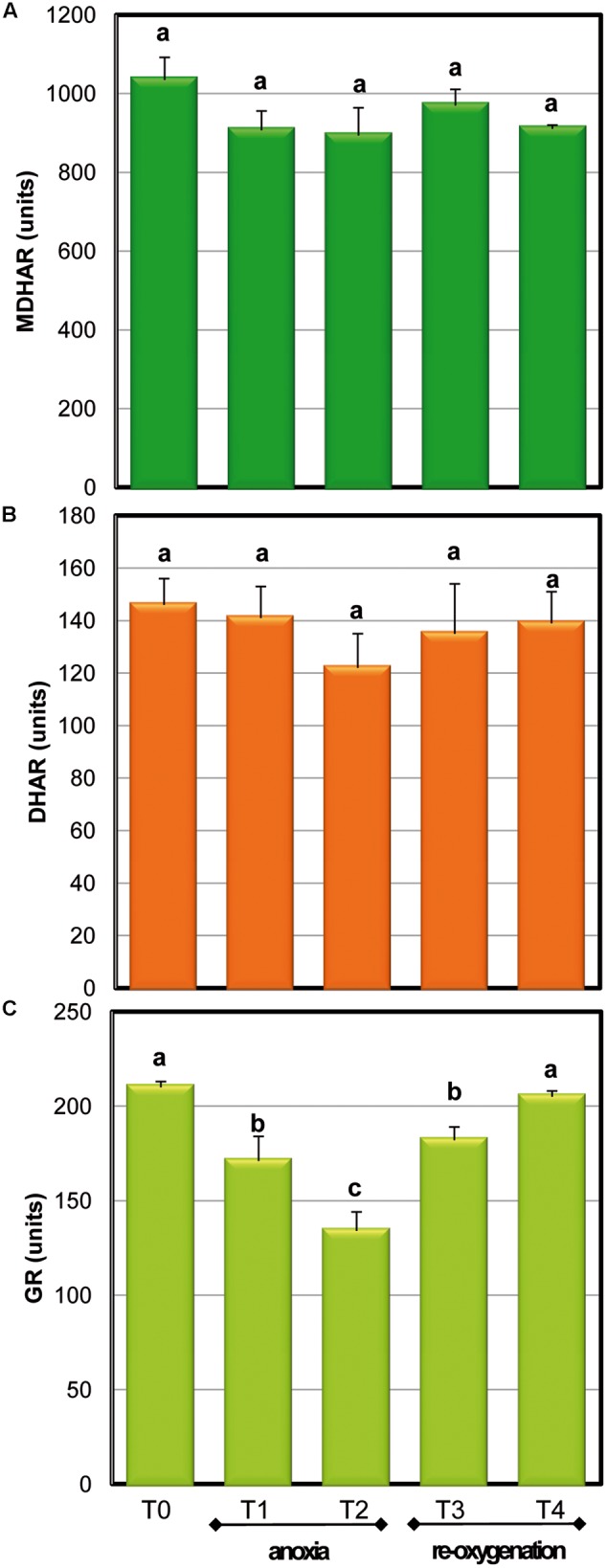
**Effects of anoxia and re-oxygenation on ASC-GSH recycling enzymes in *A. thaliana* cell suspension cultures.** Activities are expressed in units. MDHAR **(A)**: 1 unit = 1nmol NADH oxidized min^-1^mg^-1^ prot; DHAR **(B)**: 1 unit = 1nmol DHA reduced min^-1^mg^-1^ prot; GR **(C)**: 1 unit = 1nmol NADPH oxidized min^-1^mg^-1^ prot. Data represent the means (±SE) of five experiments. Different letters represent values which are statistically different (by one-way Anova test).

To maintain the redox state and to regenerate antioxidant molecules in their active form, several enzymes act to support the antioxidative defense. The recycling enzymes of ASC-GSH cycle were tested in order to follow their behavior in *Arabidopsis* cell cultures at different oxygen concentrations (**Figure 5**). MDHAR and DHAR, the enzymes responsible for the reduction to ASC of MDHA and DHA, respectively, did not change significantly in response to both limited oxygen availability and following re-oxygenation. The activity of GR, enzyme responsible for glutathione reduction, significantly decreased during anoxia and gradually reached control values after re-oxygenation. It is worth noting that in spite of GR decreases during anoxia, the redox state of GSH pool remained high, thus suggesting that GR activity was overabundant.

**FIGURE 6:**
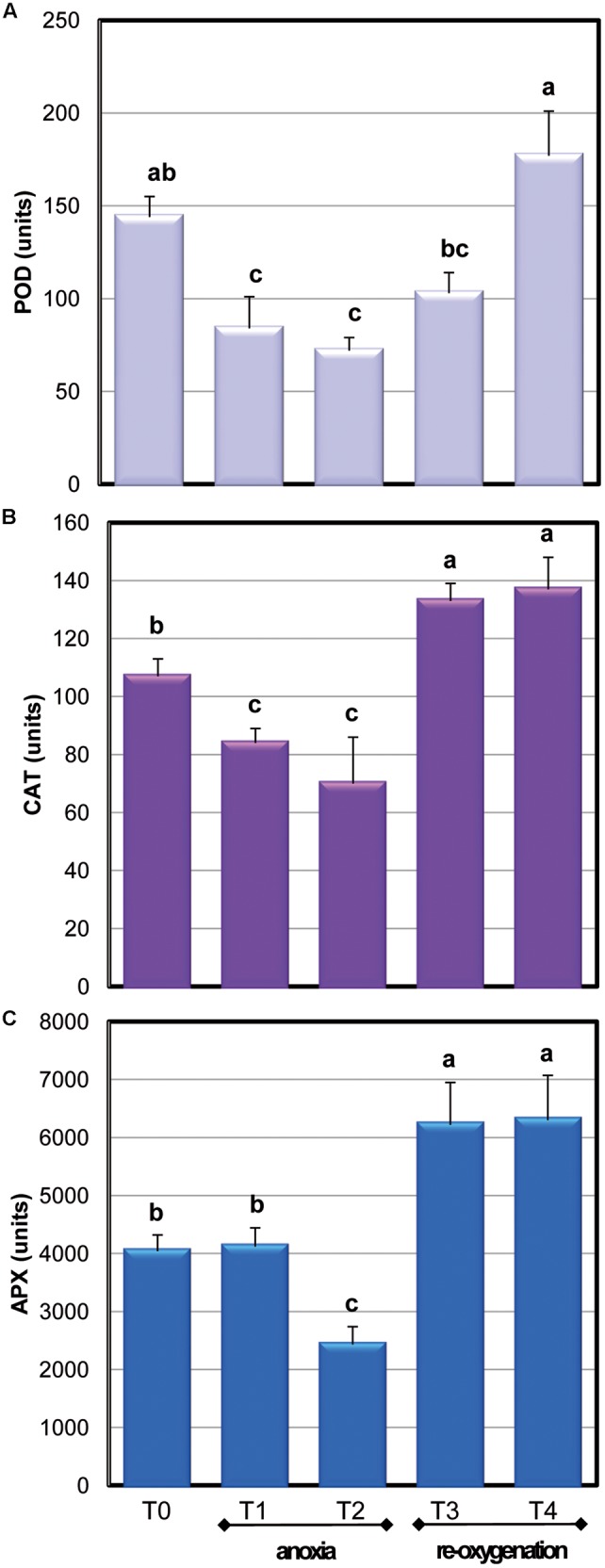
**Effects of anoxia and re-oxygenation on antioxidant enzymes in *A. thaliana* cell suspension cultures.** Activities are expressed in units. **(A)** APX: 1 unit = 1nmol ASC oxidized min^-1^mg^-1^ prot; **(B)** CAT: 1 unit = 1nmol H_2_O_2_ dismutated min^-1^mg^-1^ prot; **(C)** POD: 1 unit = 1nmol TMB oxidated min^-1^mg^-1^ prot. Data represent the means (±SE) of five experiments. Different letters represent values which are statistically different (by one-way Anova test).

To obtain information of the global H_2_O_2_ removal capability of *Arabidopsis* cells in response to oxygen concentration changes, the activity of APX, CAT, and POD was also measured (**Figure 6**). POD and CAT activity drastically decreased during anoxia to about 50% of the control value already after 2 h of oxygen deprivation; re-oxygenation induced a quick restoration of CAT activity while POD activity was completely restored only at the end of the experiment.

**FIGURE 7:**
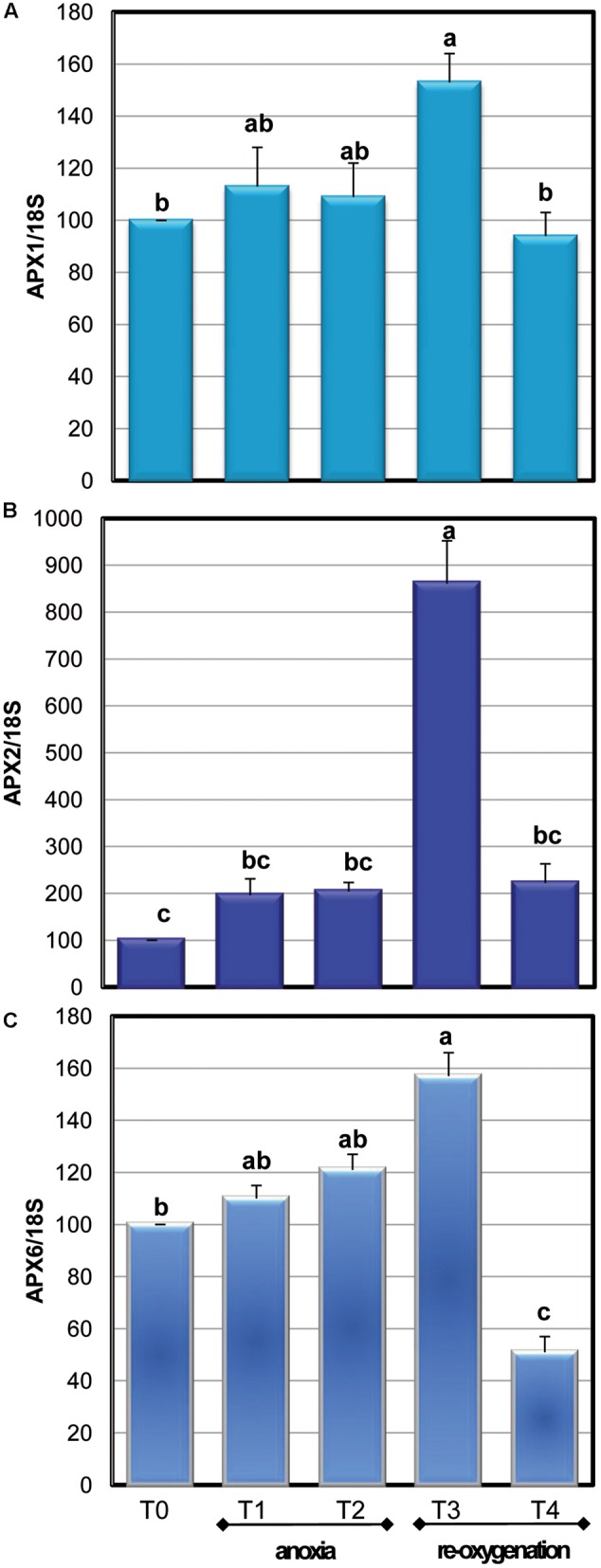
**Effects of anoxia and re-oxygenation on the expression of *APX1* (A)**, *APX2* **(B)**, and *APX6* **(C)** in *A. thaliana* cell suspension cultures. Gene expression changes were measured by RT-PCR using 18S rRNA as the internal control. Different letters represent values which are statistically different (by one-way Anova test).

Among the APX isoenzymes, the activity of cytosolic ones (cAPX) was determined: under our experimental conditions, photosynthesis was not active (data not shown). Moreover, cytosol has been reported to act as key site of redox signaling integration (Noctor and Foyer, 2016). Anoxia resulted in a significant 40% decrease of cAPX activity only at T2. The high activity of this H_2_O_2_ scavenging enzyme during the first period of oxygen deprivation supports a key role for this enzyme in overcoming oxidative damage. During re-oxygenation, cAPX activity rapidly increased. This result is coherent with other evidences obtained when oxygen-deprived roots of wheat seedlings were re-aerated (Biemelt et al., 1998). Three genes encoding for cAPXs are present in *Arabidopsis* (Noctor et al., 2016). To test whether alteration in cAPX occurring during anoxia and re-oxygenation was due to gene expression changes, the transcript accumulation of these three genes was analyzed (**Figure 7**). The sequences of RT-PCR amplifications were compared to predicted mRNA sequences, confirming the correspondence with *Arabidopsis* genes (data not shown).

During anoxic treatment, the three c*APX* genes exhibited a similar pattern of transcript accumulation, showing no significant changes in their expression. Re-oxygenation induced an immediate increase in the transcript accumulation that was particularly high for APX2. This is consistent with the pivotal role of APX2 in responses to oxidative stress, since this isoenzyme is considered a stress marker and its expression is induced by several kinds of stresses (Gallas and Waters, 2015). Redox-regulated transcription factors have been characterized. The promoter of *Arabidopsis APX1* and *APX2* contains a heat shock element binding a heat shock factor sensitive to endogenous H_2_O_2_ production (de Pinto et al., 2015). In rice, an increase of H_2_O_2_ levels enhanced the expression of two cytosolic *APX* genes (Li et al., 2015). For all the three analyzed c*APX*s the expression values returned comparable to the control after 20 h of re-oxygenation. The increase in c*APX* gene expression observed when the anoxic cells were again exposed to oxygen might be related to the increase of enzyme activity occurring during re-oxygenation. On the other hand, no correlation was observed between the decrease of cAPX activity occurring at T2 and gene expression changes. It has been suggested that post-translational modifications are rapid regulators of enzyme activity. Most of these post-translational modifications are redox regulated and involve NO or ROS, such as carbonylation of specific amino acids, thiol – disulphide transition and *S*-nitrosylation (de Pinto et al., 2013; Waszczak et al., 2015). Due to the strong oxidative environment occurring during oxygen deprivation, it is likely that redox post-translational modifications are responsible for changes in enzyme activities, even if more in deep studies are required for verifying this hypothesis.

## Conclusion

The results obtained in the present study using *Arabidopsis* suspension cell cultures grown in a stirred bioreactor indicate that imbalance of cell redox state occurs during the anoxic shock due to the dramatic increase of ROS, H_2_O_2_, and NO. On the other hand, the evaluation of cell defenses during anoxia and re-oxygenation and the absence of cell death suggest that the overproduction of reactive species triggers signaling pathways activating the antioxidant machinery such as ASC-GSH system, α-tocopherol and the antioxidant enzymes APX, CAT, and POD. The enhancement of these ROS-scavenging/controlling systems leads cells to counteract the stress toward cell survival, probably cooperating with other mechanisms controlling ROS production and scavenging to cope with the metabolic impairment occurring during anoxia (Pucciariello and Perata, 2016). This study also indicates that bioreactors can be a useful tool for studying cell responses to qualitative and quantitative changes of chemical and physical parameters affecting cell life, thus opening new opportunities for monitoring the impact of environmental changes on cell metabolism.

## Author Contributions

AP performed biochemical and molecular analyses and contributed to drafting the manuscript. SC designed and coordinated the experimental work; wrote the manuscript. AL designed and conducted confocal microscopy analyses; contributed to drafting the manuscript. AB performed biochemical analyses. RN performed cell culture experimental work and HPLC analyses. LD analyzed and interpreted data; critically revised the manuscript for important intellectual content.

## Conflict of Interest Statement

The authors declare that the research was conducted in the absence of any commercial or financial relationships that could be construed as a potential conflict of interest.

## Abbreviations

APX: ascorbate peroxidase
ASC: ascorbate
carboxy-H_2_DCFDA: 5-(and-6)-carboxy-2′,7′-dichlorodihydrofluorescein diacetate
CAT: catalase
DAF-2 DA: 4,5-diamino-fluorescein diacetate
DHAR: dehydroascorbate oxido-reductase
DHR: dihydrorhodamine 123
GPX: glutathione peroxidase
GR: glutathione reductase
GSH: reduced glutathione
GSSG: oxidized glutathione
MDHAR: monodehydroascorbate reductase
NO: nitric oxide
POD: peroxidase
ROS: reactive oxygen species
